# Assessing the use of social media in physician assistant education

**DOI:** 10.5116/ijme.5c14.ef82

**Published:** 2019-01-29

**Authors:** Gregory K. Wanner, Andrew W. Phillips, Dimitrios Papanagnou

**Affiliations:** 1 Department of Emergency Medicine, Christiana Care Health System, Newark, USA; 2 Department of Emergency Medicine, University of North Carolina, Chapel Hill, USA; 3 Department of Emergency Medicine, Sidney Kimmel Medical College at Thomas Jefferson University, USA

**Keywords:** Social media, physician assistant, education, medical education, technology

## Abstract

**Objectives:**

This study aims to assess physician assistant (PA) students’ experiences with social media (SM) as a part of their medical education.

**Methods:**

The study is split into two phases: Phase 1- A cross-sectional survey emailed to all PA students at four PA school campuses to assess students’ prior SM experiences (226 responses, 71.1% response rate); and Phase 2- Inclusion of SM educational resources, via Twitter, within lectures performed at two PA schools. A phase-2 survey assessed students’ opinions of educational SM (50 responses, 59.5% response rate) and SM usage was tracked.

**Results:**

The phase-1 survey respondents indicated that 97.3% (n=220) use social media; often used as a part of their education, 65% (n=147) informally and 2.7% (n=6) formally incorporated. Students most commonly use Facebook, YouTube, and Instagram, but rarely use Twitter. Currently using SM for medical education was significantly associated with predicting that future PA education will formally include SM [r_s_=.341 (r^2^=0.12), p=<.001], as did younger age, [r_s_=.137 (r^2^=0.02), p=0.042]. Of phase-2 survey respondents, 93.1% (27/29) of SM users felt it was a useful addition to the lectures. Significantly more views were captured when messages were sent during lectures Mean (SD), 102.64(39.7) than in the peri-lecture time period [49.5(10.6), p<0.001].

**Conclusions:**

Many PA students are currently using various forms of social media to augment their education. Most PA students support formal incorporation of social media into their education.  PA educators should consider using our data and methods of social media inclusion when designing curricula and while clinically precepting PA students.

## Introduction

The majority of today’s physician assistant (PA) students have been immersed in internet-based technologies since early childhood.^1^ Social media (SM) is an extension of those technologies. The term “social media” encompasses several aspects of electronic communication and collaboration including blogs (e.g., Blogger), micro-blogs (e.g., Twitter), social networking (e.g., Facebook), video-sharing (e.g., YouTube, Snapchat), and photo-sharing (e.g., Instagram).^2^^-^^4^ Unlike traditional written media, such as textbooks, social media is interactive, allowing users to add comments or content. In recent years social media has become an increasingly larger part of health care education.^3^^,^^5^ Several studies have evaluated the use of social media in health care educational programs. Medical students, nursing students, pharmacy students, and resident physicians in several specialties use social media resources as formal and informal parts of their education.^3^^,^^4^^,^^6^^-^^10^ A 2016 systematic review indicated nursing students use social media to “enhance students’ confidence” and engage in peer-learning.^7^ Resident physicians appear to use social media—particularly Twitter, podcasts, and blogs—to enhance their education through the rapid dissemination of evidence-based information, according to a recent systematic review.^2^The concept of “Free Open Access Medical education (FOAM)”, or freely available educational resources available via social media, has become popular among emergency medicine residents and physicians on Twitter; and often referred to as “FOAMed”.^11^^-^^13^Several studies have also evaluated social media use in the education of medical students. Four studies involving medical students incorporated Twitter into existing courses, including a clinical medicine course, neuroanatomy course, ultrasound training elective, and surgery clerkship.^14^^-^^17^ The studies suggested increased communication and idea sharing, improved morale, and positive opinions from students.

Traditional educational resources, such as lectures, textbooks and journal articles, are the mainstay of PA and medical education. Experienced clinicians and educators typically feel comfortable with these teaching modalities, and students continue to learn using these methods.^1^ However, with the electronic age, new concepts in education have emerged, including “blended learning” and the “flipped classroom”. The idea of blended learning combines different learning modalities for a given topic, such as traditional face-to-face learning with asynchronous online learning experiences.^18^^,^^19^ The flipped classroom model assigns homework as asynchronous online videos or audio podcasts to prepare for active learning sessions in the classroom.^1^ Social media can work with these new concepts to provide an electronic adjunct to traditional educational methods. Additionally, for practicing clinicians, social media can improve the knowledge translation of new treatments or techniques.^3^^,^^13^^,^^20^

Students in many healthcare-related fields appear to use social media resources as formal and informal parts of their educational programs. Social media and electronic resources have been blended into these programs as an adjunct to a classroom and clinical education. Little is known, however, about the use of social media in physician assistant education.

This study aimed to establish local PA students’ use of social media in their personal lives and their education and to assess students’ experiences and interactions with social media introduced as a formal part of their medical education.

## Methods

### Study design and participants

The study consisted of two phases. In phase 1, a cross-sectional survey of all first and second-year PA students at four program campuses (3 main campuses and 1 branch campus) was conducted to measure students’ opinions of and prior experiences with social media related to their PA education. Phase 1 was assessed with the “Phase 1 survey”.  In phase 2, a longitudinal observation survey was conducted after incorporation of social media educational resources into two first-year PA classes. Students’ opinions concerning the use of social media as a part of their education were assessed with the “Phase 2 survey”.

### Data collection

First and second year PA students at four PA school campuses in the northeastern United States were eligible. Only current physician assistant students were eligible for inclusion. PA faculty and graduates were excluded from this study. Data was collected through surveys sent by email and by tracking anonymous Twitter usage statistics. Demographic data of respondents for phase 1 and phase 2 surveys are reported in Table 1.

**Table 1:** The participant demographics (Phase-1, N=226; Phase-2, N=50)

Participants		Phase 1 Survey		Phase 2 Survey	
		%	n	%	n
Gender					
	Female	75.2	170	74	37
	Male	24.3	55	24	12
	Other/prefer not to answer	0.4	1	2	1
Age Category (years)					
	18-23	19.8	45	20	10
	24-29	63.4	144	72	36
	30-35	11.5	26	4	2
	36-41	3.5	8	2	1
	>42	1.3	3	2	1
PA Student Status					
	First year	39.8	90	100	50
	Second year	60.2	136	0	0

The phase 1 survey included 14 questions to assess demographics of participants, current usage of social media in their personal lives and related to their medical education, their primary sources for medical education, and their opinions regarding social media incorporation into their educational program (rated on a 5-point Likert scale). The phase 1 survey was sent to 318 students and responses were received from 226 students (response rate of 71.1%). The phase 2 survey included 11 questions to assess demographics, participants’ prior usage of social media in their medical education, and their opinions and experiences regarding the incorporation of social media resources into their classwork (rated on a 5-point Likert scale). The phase 2 survey was sent to 84 students, and 50 students responded (response rate 59.5%). Students were also able to provide comments in the surveys. Both surveys were created based on an extensive literature review and were pilot tested with three stakeholders including former students and faculty. Validity was assessed using Messick’s unified framework, of which internal structure was calculated with Cronbach’s alpha.^21^

### Procedure

The study was conducted between March and June of 2016 and received an exemption status from the institutional review board at Thomas Jefferson University in Philadelphia, Pennsylvania, USA. Participation in the study was voluntary and did not influence course activities or grades. The phase 1 survey was sent by email to all PA students at the four PA school campuses with one reminder sent approximately four weeks later. After the phase 1 survey enrollment was closed, social media resources were incorporated into a pre-established gastrointestinal emergencies lecture series given at two different PA schools. The resources were in the form of 11 Twitter messages (“tweets”) sent by the lecturer during or immediately after the lectures from a Twitter account specifically created for the study. The tweets/messages included clarification points, review slides produced by the lecturer, and web-links to reputable blog articles or videos related to the lectures. The material sent was pre-selected by the lecturer. Students were encouraged to use the social media resources outside of class to augment their in-class lecture experience. Use or non-use of the resources had no bearing on their grade for the course. The number of views (referred to as “impressions” on the Twitter platform) of each tweet was followed, and the total number of views were recorded five-days after the message was posted. Twitter Analytics was used to track the number of views for each tweet. To help control for background views/impressions (those not related to PA students in the specified lectures), a total of six “control” messages were sent; three were sent several weeks prior to the lectures, and three sent starting nine days after the final lecture. The phase 2 survey was sent by email at the conclusion of the course to students at the two programs where social media was formally incorporated into their classwork. Two reminders were sent for the phase 2 survey.

**Table 2:** Phase 1 survey responses regarding social media use (N=226)

Survey Item		%	n
Social media use			
	Not at all	2.7	6
	Only for personal use	29.6	67
	Informal part of PA education, on own	65.0	147
	Formally included part of PA education	2.7	6
Current social media accounts (n=226)			
	Facebook	97.3	220
	Instagram	65.0	147
	LinkedIn	43.4	98
	Google+	33.2	75
	Twitter	22.1	50
	Other (Snapchat, Figure 1, etc)	7.1	16
	MySpace	0.9	2
Social media used for education (n=162^*^)			
	Facebook	69.8	113
	YouTube	56.2	91
	Other^†^	34.6	56
	Medical blogs	13.6	22
	Google+	9.3	15
	Twitter	1.2	2

*Includes only the respondents who indicated that they use social media as a part of their education †Instagram was most frequently reported in this category

### Validity evidence for the survey instruments

Following Messick’s framework, the content validity was established by stakeholder evaluation. Feedback from the pilot group provided a modified think-aloud analysis that did not reveal any observation/record discrepancies. Internal structure and reliability were supported with acceptable Cronbach’s alphas (phase 1 survey Cronbach’s alpha, 0.916; phase 2 survey Cronbach’s alpha, 0.778). Internal structure and reliability are slightly different for the phase 1 and phase 2 surveys, but answers were not compared between the two surveys, and both values are considered strong for validity evidence, having crossed a binomial threshold for acceptable use to evaluate the primary outcome. Additionally, predicted relationships existed between variables, notably, the significant correlation between those using social media having greater support for it. Finally, the requirement for the theoretical consequences of the responses is met by our positive findings in the context of previously published studies.^14^^-^^17^

**Table 3:** Phase 2 survey responses regarding social media use in PA education (N=50)

Survey Item		%	n
Used social media resources related to lectures			
	Yes	58	29
	No	42	21
Most likely to use, if incorporated into PA education			
	YouTube	58	29
	Facebook	56	28
	Instagram	52	26
	Medical blogs	32	16
	Twitter	22	11
	Medical image websites	20	10
	Podcasts	18	9
	Google+	10	5
useful way to incorporate into PA education			
	Clarify lecture topics	63.3	31
	Additional educational resources	63.3	31
	Extra credit assignments	42.9	21
	Method of self-learning	28.6	14
	Graded assignments	2	1
	Should not be included	2	1

### Statistical analysis

Descriptive and univariate statistics were performed with SPSS v24 (IBM Corporation, NY). Mann-Whitney U, Spearman’s ρ and χ^2^were used as appropriate. Alpha <0.05 was considered statistically significant.

## Results

### Phase 1 Survey

Of the 226 respondents, nearly all have at least one social media account, most commonly Facebook followed by Instagram (Table 2). Regarding the use of social media, 65.0% (n=147) of the 226 respondents report using social media as an informal part of their education whereas 2.7% (n=6) report formal inclusion as part of their education. Only 2.7% (n=6) of respondents do not use social media at all. Facebook was the most commonly used social media educational resource followed by YouTube (Table 2).

Of 220 respondents, 50.5% (n=111) somewhat or strongly agreed that social media resources should augment the existing PA curriculum, while 32.7% (n=72) neither agreed nor disagreed, and 16.8% (n=37) somewhat or strongly disagree. Of 223 respondents, 69.5% (n=155) somewhat or strongly agreed that they support social media use in PA education with only 10.3% (n=23) somewhat or strongly disagreeing. Additionally, 50.5% (n=112) of 222 respondents strongly or somewhat agreed that social media resources will be formally included in PA education in the future, while 33.3% (n=74) neither agreed nor disagreed. Results are reported in Table 4.

**Table 4:** Opinion-based responses regarding social media use in PA education (Phase-1, N=226; Phase-2, N=50)

Phase and Statement	Rating	
	Mean	SD
Phase 1 Survey		
Support SM inclusion in PA education (n=223)	2.23	0.98
Should augment existing PA curriculum (n=220)	2.56	1.01
SM will be formally included in the future (n=222)	2.53	1.01
Phase 2 Survey		
Support SM inclusion in PA education (n=49)	1.96	0.78
Worthwhile addition to the lectures (n=33)	1.94	0.85
Useful addition to PA curriculum (n=50)	2.06	0.83

Respondents were also asked to rank their primary sources of information for their education from most used (rating of 1) to least used (rating of 4). Textbooks [mean (SD), 1.68 (0.775)] and electronic resources such as UpToDate or Epocrates [1.68 (0.729)] were the most highly ranked study tools, followed by journal articles [3.01 (0.696)] and blogs or social media [3.62 (0.735)]. Currently using blogs or social media for formal or informal medical education was significantly associated with predicting that future PA education will formally include social media, [r_s_=.341 (r^2^=0.12), p=<.001], as did younger age [r_s_=.137 (r^2^=0.02), p=0.042].

In addition to numerical ratings, respondents were asked to provide written comments regarding social media use in PA education. Thirty-five students provided comments. Most comments fell within a few general categories: How social media is used (16 comments), questioning sources/finding reliable information (10 comments), potential as a distraction (4 comments), positive opinions (9 comments), and negative opinions (3 comments). Many respondents felt that social media resources should be an optional part of PA education. Examples of specific comments include:

“Social media is good for students who are adept at critically evaluating a source. It becomes dangerous when students do not have a background in determining a sound source.” (Second-year PA student, female, age range 30-35 years)

“Good for introducing a topic through a platform that students often use…[but] I do not feel students should rely solely on social media.” (Second-year, female, age range 24-29 years)

“It may be difficult to get most students to take the time to check out the extra resources if they already have such as heavy course load.” (First-year, female, age range 18-23 years)

“My one concern with social media is that there are so many resources out there…having guidance from professors on the sources to go to would be helpful.” (First-year, female, age range 24-29 years)

### Intervention

The number of views for each message posted on Twitter were tracked after the introduction of social media resources into the lecture series. A significant increase in the number of views/impressions was observed when messages/tweets were sent during the lecture period [mean (SD), 102.64 (39.7)] than in the peri-lecture “control” time period [49.5 (10.6)], p <0.001 by Mann-Whitney U.

### Phase 2 Survey

Of the 50 responses, PA students report that they would be most likely to use YouTube (n=29), Facebook (n=28), and Instagram (n=26), if incorporated into their education (Table 3). Students felt the most useful ways to integrate social media include “clarify topics presented in lecture” and “provide resources for education outside of the classroom” (Table 3). When students were asked if social media would be a useful addition to their current PA curriculum, 78% (39 of 50) strongly or somewhat agreed that social media would be a valuable addition to their current PA curriculum, 14% (n=7) neither agreed nor disagreed, and 8% (n=4) somewhat disagreed. However, those students who used social media during the course (58%, 29 of the 50 phase 2 survey respondents) agreed significantly more that it would be a useful addition to their curriculum (p=0.027 by Mann-Whitney U). Fully 93.1% of those who used social media during the course (27 of 29) strongly or somewhat agreed that it was a useful addition, compared to 57.1% of those who did not use social media during the course (12 of 21). After the course, students were asked whether they support the inclusion of social media in their educational coursework. Of 49 question responses, 79.6% (n=39) strongly or somewhat agreed, 16.3% (n=8) neither agreed nor disagreed, and 4.1% (n=2) somewhat disagreed with the statement of support for including social media in the course. Additional results are reported in Table 4.

## Discussion

This study aimed to assess PA students’ current use of social media and their opinions related to the formal inclusion of social media resources into their coursework. According to our study, most physician assistant students are already using social media as an informal part of their PA education. PA students most commonly use Facebook (including class-related Facebook “groups”) followed by YouTube and Instagram. While most students support the idea of formally incorporating social media into their PA education, many felt it should be an optional adjunct to their classroom and clinical education. Integrating Twitter into an existing lecture series resulted in a significant increase in the number of messages/tweets viewed. Despite our evidence that PA students rarely use Twitter, most students viewed and participated in the social media resources made available on Twitter. This indicates a high-interest level and willingness to participate among PA students. Students also had a positive opinion of this method of social media inclusion. Students who used social media resources during the course were much more likely to find the method useful, compared to students who did not use the resources.

Several students identified concerns about using social media, including finding reputable and reliable resources. For our study, each of the blog links and YouTube videos were reviewed by the lecturer to ensure accurate and reliable content. Students should be instructed on methods of finding reputable sources of social media. Indicators of quality social media sources include identified authors with stated qualifications and conflicts of interest, clear distinctions between fact and opinion, cited references, accurate information, and transparency about who was involved in the creation of the resource.^22^ In addition to ensuring reputable sources, additional concerns such as professionalism and privacy should be considered when using social media for educational purposes.

Several articles have suggested potential professionalism issues with social media, including inappropriate posts or photos depicting unprofessional or potentially illegal activities.^23^^-^^25^ Examples of unprofessional posts include disclosure of identifiable patient information in photos and radiographs or images of intoxicated colleagues.^23^^,^^25^ Some of these cases have resulted in disciplinary action. As such, incorporation of social media into an educational program should be coupled with a discussion of appropriate use, patient privacy, and professionalism.

Despite these concerns, the majority of surveyed students support the formal inclusion of social media into their education and felt that our methods were useful. As was seen in this and other studies, the use of social media resources can help increase students’ participation and interest with a modality that is familiar to them. Social media can also mesh well with newer concepts in education, such as the “flipped classroom” or “blended learning”. While most students continue to use textbooks as their primary source of educational information according to our study, users of social media foresee social media becoming formally included into PA education in the future, as has been demonstrated in other aspects of health care education.^2^^,^^7^^,^^14^^-^^17^ PA educators should consider this while designing course content and curricula. Additionally, preceptors of PA students should consider social media resources as a method to connect with, engage, and educate PA students, many of whom are already using these resources.

### Limitations

We used a survey to evaluate student’s opinions of and experiences with social media. Opinion-based surveys are intrinsically subjective and can be limited by recall bias. While our response rates were fairly high and surveys were sent to several different PA schools, all schools and branch campuses were located in the northeast region of the United States, potentially limiting the generalizability of results across all PA schools. We incorporated Twitter into a lecture series based on data and experiences in medical and residency education. It appears, however, that few PA students regularly use Twitter, instead favoring Facebook, Instagram, and YouTube. Quantifying the positive or negative effects of social media on exam scores, clinical performance, or knowledge retention was beyond the scope of this study but are important topics for future research. Further studies should also focus on determining the best methods of incorporating social media into PA education, which social media platform may be most effective, and evaluating the opinions of faculty and practicing PAs regarding the use of social media.

## Conclusions

We aimed to assess how PA students use social media in their personal lives and their education. We showed that many physician assistant students are already using several forms of social media as an informal part of their medical education. We incorporated social media into an established lecture series and assessed students’ opinions and usage of the included resources. Our study indicated a high-interest level and willingness to participate among surveyed students. Interestingly, PA students also identified difficulties in finding reputable sources of educational social media and sought guidance on this topic. Future studies should focus on whether social media use affects exam scores or knowledge retention, which was beyond the scope of our study. To help engage students, PA educators and clinically-practicing PAs should consider using our data and methods of social media inclusion when designing curricula and while clinically precepting PA students.

### Conflict of Interest

The authors declare that they have no conflict of interest.
